# Supplementary material for: Differences in natriuretic peptide response in self-identified white and black individuals: a physiological clinical trial
Source: Nat Commun. 2025 Feb 13;16:1621. doi: 10.1038/s41467-024-55648-2 (PMC11825667; doi:10.1038/s41467-024-55648-2)
Supplement: Supplementary file 1 — Supplementary Information [file 41467_2024_55648_MOESM1_ESM.pdf]

Differences in Natriuretic Peptide Response in Self-Identified White and Black Individuals: A Physiological Clinical Trial

Naman S. Shetty[1,2], Mokshad Gaonkar[3], Nirav Patel[3], Nehal Vekariya[3], Krishin Yerabolu[3], Jasninder S. Dhaliwal[3], Thomas W. Buford[4,5], Barbara Gower[6], Peng Li[7], Thomas J. Wang[8], Garima Arora[3], Pankaj Arora[3,9]

Affiliations

1. Department of Anesthesia, Critical Care and Pain Medicine, Massachusetts General Hospital, Boston, MA
2. Harvard Medical School, Boston, MA
3. Division of Cardiovascular Disease, University of Alabama at Birmingham, Birmingham, AL, USA.
4. Department of Medicine, University of Alabama at Birmingham, Birmingham, AL, USA.
5. Birmingham/Atlanta VA GRECC, Birmingham Veterans Affairs Medical Center, Birmingham, AL, USA.
6. Department of Nutrition Sciences, University of Alabama at Birmingham, Birmingham, AL, USA.
7. School of Nursing, University of Alabama at Birmingham, Birmingham, AL, USA.
8. Department of Medicine, University of Texas-Southwestern Medical Center, Dallas, Texas, USA.
9. Section of Cardiology, Birmingham Veterans Affairs Medical Center, Birmingham, AL, USA.

Supplementary Methods

Interested participants would be contacted telephonically to ascertain eligibility. Eligible participants would be scheduled for a screening visit at the clinical research unit (CRU) at UAB. During the visit, the study protocol was explained to the participants and they completed questionnaires on their medical and family history. Participants who provided their consent to participate in the study were evaluated by the study investigators. Blood was drawn for a complete blood count, basic metabolic profile, and liver enzymes. All female participants underwent a urine pregnancy test at screening and each follow-up visit. The CRU nursing staff measured each participant's blood pressure (average of three readings, each taken 5 minutes apart after 15 minutes of rest), height, and weight. The participants also met the study dietician at the end of the visit. The dietician discussed the 3-day salt-standardized study diet to be consumed before the exercise challenge with the participants.

Three days of standardized meals were provided to be consumed for the 3 days before the exercise challenge visit. Three meals and snacks were provided per day based on the participant's preferences and required daily allowances as per the 2005 USDA Guidelines. Briefly, the daily diet was composed of 51% carbohydrate, 31% fats, 18% proteins, ~4.5 gm sodium, and ~2.5 gm potassium. The participants were requested to consume only the meals provided during the 3-day period and refrain from consuming any drink other than water.

Due to logistical limitations for conducting clinical research visits during the COVID-19 pandemic, 12 participants completed the medication phase before the exercise phase of the study while the remaining 68 participants completed the protocol as planned. Among the 12 participants completing the medication visit first, the median duration between the end of the medication visit and the exercise challenge was 8 (6, 15) days. Among the 68 participants

completing the exercise visit first, the median duration between the exercise challenge and the first visit of the metoprolol phase of the study was 22 (13, 31) days.

Supplementary Note 1

Study Design

This was an investigator-initiated single-center clinical trial conducted at the University of Alabama at Birmingham. All participants provided written informed consent. The study was approved by the University of Alabama at Birmingham Institutional Review Board. The detailed study protocol is included in this document. This study was registered on ClinicalTrials.gov as NCT03070184.

Rationale and Hypothesis

There are significant racial disparities in the burden of cardiovascular disease, with hypertension and heart failure being more prevalent in Black individuals than in White individuals. We have observed lower resting levels of NPs in Black individuals, raising the possibility of impaired NP production. Little is known about whether these race-related differences extend to dynamic changes in NP levels. Beta-blockers are commonly used drugs in hypertension, especially in individuals with co-existing coronary artery disease. They are also a cornerstone of standard heart failure therapy. Beta-blockers have been shown to be associated with a short-term increase in NPs. Hence, we hypothesize that Black individuals will have reduced release of NPs after 6 weeks of metoprolol compared with White individuals.

Similarly, exercise has been shown to increase the plasma concentration of NPs immediately after peak exercise in multiple previous human studies of healthy volunteers.[73,80,81,97] Although the mechanism is incompletely understood, it is thought that exercise causes degranulation of secretory granules located within the heart.[75,98] The amount of NPs stored within the secretory granules is known to rise in parallel to the resting concentrations of NPs.[99-102] As mentioned above, NP values have been shown to be lower in Black individuals compared with their White counterparts. Therefore, Black individuals may have a lower NP response to exercise compared with White individuals.

Specific Aims

The proposed single-center clinical trial will compare the response of the NP system to metoprolol and exercise among healthy, young Black and White individuals.

Primary Aim

To compare the NP (NT-proBNP, BNP, and MR-proANP) response with metoprolol therapy at 6 weeks in Black and White individuals.

Secondary Aim

To compare the NP (NT-proBNP, BNP, and MR-proANP) response immediately after standardized exercise in Black and White individuals.

*Participant Selection Inclusion Criteria***:**

1. Self-identified Black or White adults
2. Age between 18 and 40 years
3. Body mass index between 18 to 30 kg/m^2
4. No history of hypertension (seated blood pressure <140/90 mm Hg and not taking antihypertensive medications)
5. Willing to take the study medication
6. Able to exercise

Exclusion Criteria:

1. Decreased renal function (estimated glomerular filtration rate <60 $mL/min/1.73m^2$)
2. Systolic blood pressure <100 mm Hg, diastolic blood pressure <60 mm Hg, or heart rate <60/min at screening
3. Use of antihypertensive medications
4. History of cardiovascular disease
5. History of diabetes or use of antidiabetic medications
6. Anemia (hematocrit <41% in males and <35% in females)
7. Pregnancy (using urine pregnancy test)
8. Hormone replacement therapy/oral contraceptive use
9. Elevated liver function tests (>3x upper normal limit)
10. Depression
11. History of smoking or current smoking

Recruitment

The proposed single-center clinical trial will be conducted at the University of Alabama at Birmingham (UAB). UAB is located in Birmingham, AL (metropolitan population ~ 1.2 million: 29% African-American, 56% white), making this an ideal environment in which to conduct studies that include African-American and white individuals. Several recruitment approaches will be used: 1) advertisements will be posted in local newspapers and online to recruit healthy subjects from the greater Birmingham area; 2) announcements regarding the study will be made on radio stations that have high market penetration in African-American communities to enhance recruitment; and 3) outreach through the UAB CCTS Community Engagement Research Core.

Study Procedures

A participating subject's study period will span ~13-16 weeks from the screening visit to study completion. The timeline and study procedures are shown in **Supplementary Table 1**.

Supplementary Table 1. Study Flowchart and Timeline

Days	Days from Previous Visit	Visit	Location	Time (hours)	Procedures
-10 to -8	0	Pre-Screening	Home	0.5	-Discuss study objectives, inclusion/exclusion criteria, timeline, procedures & possible side effects<br>- Telephonic pre-screening
-7	0	Screening Visit (Visit 1)	CRU	1	-Written consent<br>-History & physical exam<br>-Vitals check<br>-Screening Labs<br>-Urine Pregnancy Test (if applicable)<br>-Dietician meeting (to review the standardized meals and for meal instructions)
-6	2	Exercise Capacity Determination Visit (Visit 2) + 3 days Meal pick up	UCEM	2	-Vitals Check<br>-VO2 max test<br>- 3 days meal pick up from CRU
The participants will consume 3 days of standardized meals prior to visit 3					
-3	4	ercise Challenge Test(Visit 3)	UCEM	3	-24-hour urine collection prior to the visit<br>-Vitals check<br>-Blood draw & spot urine collection<br>-Exercise Challenge test
0	1	Initiation Visit (Visit 4)	CRU	1	-24-hour urine collection prior to the visit<br>-History & physical exam<br>-Vitals check<br>-Blood draw<br>-Urine Pregnancy Test (if applicable)<br>Adherence & adverse events tracking<br>- Provide the participant with 1 pre-labeled bottle (15 tablets per bottle) of metoprolol succinate XL 50 mg - Provide the participant with the medication log
7	7	Phone Interview I	Home	0.25	-Adherence & adverse events tracking
14	7	Interim Visit 1 (Visit 5)	CRU	1	-24-hour urine collection prior to the visit<br>-History & physical exam<br>-Vitals check<br>-Blood draw<br>-Urine Pregnancy Test (if applicable)

					Adherence & adverse events tracking<br>- Provide the participant with 1 pre-labeled bottle (15 tablet per bottle) of metoprolol succinate XL 100mg<br>-Provide the participant with the medication log -Pill count and drug log check
21	7	Phone Interview II	Home	0.25	-Adherence & adverse events tracking
28	7	Interim Visit 2 (Visit 6)	CRU	1	24-hour urine collection prior to the visit<br>-History & physical exam<br>-Vitals check<br>-Blood draw<br>-Urine Pregnancy Test (if applicable)<br>Adherence & adverse events tracking<br>- Provide the participant with 1 pre-labeled bottle (15 tablets per bottle) of metoprolol succinate XL 200mg<br>-Provide the participant with the medication log -Pill count and drug log check
35	7	Phone Interview III	Home	0.25	-Adherence & adverse events tracking
42	7	Final visit (Visit 7)	CRU	1	-History & Physical<br>-Vitals check<br>-Blood Draw<br>-Urine Pregnancy Test (if applicable)<br>- Pill count and drug log check
49	7	Follow-up visit (Visit 8)	CRU	1	-Vitals check
End of Study					

Abbreviation: CRU, Clinical Research Unit; UCEM: UAB Center for Exercise Medicine

Visit # 1 (Screening Visit):

During the screening visit, the study investigator(s) will obtain consent from the study subjects. After obtaining the consent of the study subjects, personal and medical history will be taken. Physical examination, including vitals (like blood pressure and heart rate), will be performed by the study investigator(s). A urine pregnancy test will be administered to the female subjects to confirm their pregnancy status. In addition to these, 25cc (5 teaspoons) of blood will be drawn for screening labs, including baseline metabolic profile, complete blood count, and liver function tests. Furthermore, the dietician will explain the 3 days of a standardized diet. If the subject agrees to adhere to the study diet, follow the study protocol, and meet the inclusion criteria, then they will be enrolled in the study.

Visit # 2 (Baseline visit-Meal Pick-up):

Subjects will come for a baseline visit (scheduled according to subjects' convenience) to the UCEM for determination of maximal oxygen capacity (VO2 max). Subjects will be asked to fast overnight (>6 hours) prior to this visit and not to drink caffeine and alcoholic beverages 24 hours prior to this visit.

- A maximal modified Bruce treadmill protocol will be used to determine aerobic fitness/VO2 max. Monitoring will consist of a 12-lead electrocardiogram and BP measurements will be taken every two minutes.
- Oxygen uptake and carbon dioxide production will be measured continuously using a TrueOne 2400 metabolic cart.
- Achievement of maximal oxygen uptake (VO2 max) was ascertained by the exercise physiologist based on standard criteria for heart rate (HR within 10 beats/min of estimated maximum), respiratory exchange ratio (RER >1.2), and plateauing of VO2/min/kg.
- After determining the VO2 max, subjects will pick up 3 days of standardized diet from the metabolic kitchen of CRU. Meals will be provided to subjects at no cost. The daily standardized diet will be composed of 51% carbohydrate, 31% fats, 18% proteins, ~4.5 gm sodium, and ~2.5 gm potassium. All meals will be prepared in the CRU metabolic kitchen. A menu and food records form included in the packet.
- A container for 24-hour urine collection will be provided. Instruction for the collection of 24-hour urine will be provided by the study coordinator.

Prior to 3rd/Exercise Challenge visit:

Three days prior to 3rd visit, subjects will consume the meals that were provided by the CRU. Subjects will be asked to collect 24-hour urine and fast overnight (>6 hours) prior to 3rd visit. Subjects will be asked not to drink caffeine and alcoholic beverages 24 hours prior to this visit. Before coming for 3rd visit, subjects will drop off their 24-hour urine sample at the Outreach Lab.

Visit # 3/ Exercise Challenge visit:

- Subjects will come to the PAC and rest for 1 hour in supine position. Medical history and vital sign measurements (blood pressure and heart rate) will be collected. After 1 hour of rest, 20cc of blood or 4 teaspoons will be drawn by a nurse for the assays. Blood samples will be tested for natriuretic peptide levels, insulin, and glucose.
- Afterward, subjects will be asked to perform the exercise challenge test. The exercise challenge is designed to give each subject comparable amounts of physiological stress. Each subject will walk at 70% of his/her VO2 max for 20 minutes.
- The exercise challenge has a 4-minute warmup starting with 3 mph at zero grade for two minutes, progressing to 3 mph at a 3% grade for 1 minute, followed by 3 mph at a 6% grade for one minute.

- Using the American College of Sports Medicine metabolic equations, treadmill grade and speed will be adjusted to attain 70% VO2 max. Each subject will walk at 70 % of their VO2 max for 20 minutes. At the end of the exercise challenge, participants will undergo a 4-minute cool-down by walking at 2 mph at 0 grade.
- Subjects will be asked to take a rest for 30 minutes. Before, immediately, 15 minutes, and 30 minutes after the exercise challenge, blood samples will be collected to measure NP levels. Participants were asked to provide urine samples to measure urinary sodium levels at the start and 30 minutes after the end of the exercise challenge.
- During the exercise challenge visit, subjects will be asked not to drink water. Subjects can drink water after completing the exercise challenge.
- A container for 24-hour urine collection will be provided. Instruction for the collection of 24-hour urine will be provided by the study coordinator.

Visit # 4/ Drug initiation visit:

- Three to seven days after 3rd visit, subjects will come for 4th visit.
- Subjects will be asked to fast overnight (>6 hours) prior to this visit and not to drink caffeine and alcoholic beverages 24 hours prior to this visit.
- Before coming for the 4th visit, subjects will drop off 24-hour urine samples at the outreach lab. A urine sample will be tested for sodium, protein, and creatinine levels.
- Subjects will come to the CRU and will be asked to take a rest for 1 hour.
- If a female is in the reproductive age group, a urine pregnancy test will be performed to rule out pregnancy. BP and heart rate (HR) will be measured and venous blood will be drawn.
- If the systolic blood pressure is >100 mmHg, diastolic blood pressure is >50 mm Hg, and heart rate is >60 bpm, then a two-week supply of 50 mg of metoprolol succinate will be provided. The local Investigational Drug Services will dispense pre-labeled bottles (15 tablets per bottle) of metoprolol succinate XL 50 mg in bulk (6 bottles for each dose). A study physician/study coordinator will obtain these pre-labeled bottles from the research pharmacy. Drug bottles will be kept in locked cabinets. A study physician will dispense each bottle to the study subjects according to the study visits. A total of 15 capsules will be dispensed at this time, which provides sufficient supply for 2 weeks plus 1 additional pill in the event that the visit cannot be scheduled exactly 2 weeks to reach the interim visit 1 (Visit #5). Subjects will be instructed to take one capsule once a day.
- Subjects will be called by the study doctor to assess medicine side effects within 7 days after this visit.
- Drug compliance and side effects will be assessed through a telephone call after 7 days of visit and a medication log.

Visit # 5/Interim visit 1:

- Two weeks after visit #4, subjects will come for this visit
- Subjects will be asked to fast overnight (>6 hours) prior to this visit and not to drink caffeine and alcoholic beverages 24 hours prior to this visit.
- Subjects will come to the CRU and will be asked to take a rest for 1 hour.
- Blood pressure and heart rate will be measured and venous blood will be drawn after 1 hour of rest. Blood samples will be tested for natriuretic peptide levels, insulin, and glucose.
- Compliance will be assessed by doing a pill count and reviewing the medication log.
- Subjects will be asked about any possible side effects from the metoprolol use in the previous 2 weeks.
- If compliant and have no side effects, subjects will be given a 2-week supply of 100 mg/day of metoprolol succinate. The local Investigational Drug Services will dispense pre-labeled bottles (15 tablets per bottle) of metoprolol succinate XL 100 mg in bulk (6 bottles for each dose). A study physician/study coordinator will obtain these pre-labeled bottles from the research pharmacy. Drug bottles will be kept in locked cabinets. A study physician will dispense each bottle to the study subjects according to the study visits. A total of 15 capsules will be dispensed at this time, which provides sufficient supply for 2 weeks plus 1 additional pill in the event that the visit cannot be scheduled exactly 2 weeks to reach the interim visit 2 (Visit 6). Subjects will be instructed to take one capsule once a day.
- Subjects will be called by the study doctor to assess medicine side effects within 7 days after this visit.
- Drug compliance and side effects will be assessed through a telephone call after 7 days of visit and a medication log.

Visit # 6/Interim Visit 2:

- Two weeks after visit #5, subjects will come for this visit. The same protocol as that for visit # 5 will be followed. Compliance will be assessed and if no side effects are reported, subjects will be given a 2-week supply of 200 mg/day of Metoprolol succinate. The local Investigational Drug Services will dispense pre-labeled bottles (15 tablets per bottle) of metoprolol succinate XL 200 mg in bulk (6 bottles for each dose). A study physician/study coordinator will obtain these pre-labeled bottles from the research pharmacy. Drug bottles will be kept in locked cabinets. A total of 15 capsules will be dispensed at this time, which provides sufficient supply for 2 weeks plus 1 additional pill in the event that the visit cannot be scheduled exactly 2 weeks to reach the final visit (Visit 7). Subjects will be instructed to take one capsule once a day.
- Subjects will be called by the study doctor to assess medicine side effects within 7 days after this visit.
- Drug compliance and side effects will be assessed through a telephone call after 7 days of visit and a medication log.

  A container for 24-hour urine collection will be provided.

Visit # 7/Final Visit:

Two weeks after visit #6, subjects will come for this visit. On this visit, subjects will drop off 24-hour urine collection at the outreach lab. The urine sample will be tested for sodium, protein, and creatinine levels. The same protocol as that for visit # 5 will be followed. Blood will be drawn. Blood samples will be tested for natriuretic peptide levels, insulin, and glucose. At the end of this visit, the subject's participation will be complete.

Visit # 8/Follow-up Visit:

One week after visit #7, subjects will come to the CRU for their follow-up visit. After 15 min of rest, blood pressure and heart rate (three times) will be measured to evaluate subjects' well-being.

Early Termination/Discontinuation Visit:

At every visit, blood pressure and heart rate will be measured. Metoprolol will be discontinued for any systolic BP< 80 mm Hg and HR < 40 beats/minute at any visit. If the subject develops intolerance or any side effects from increasing the dose of metoprolol, they will go back to the maximally tolerated last dose to finish the 6-week duration.

Study Design

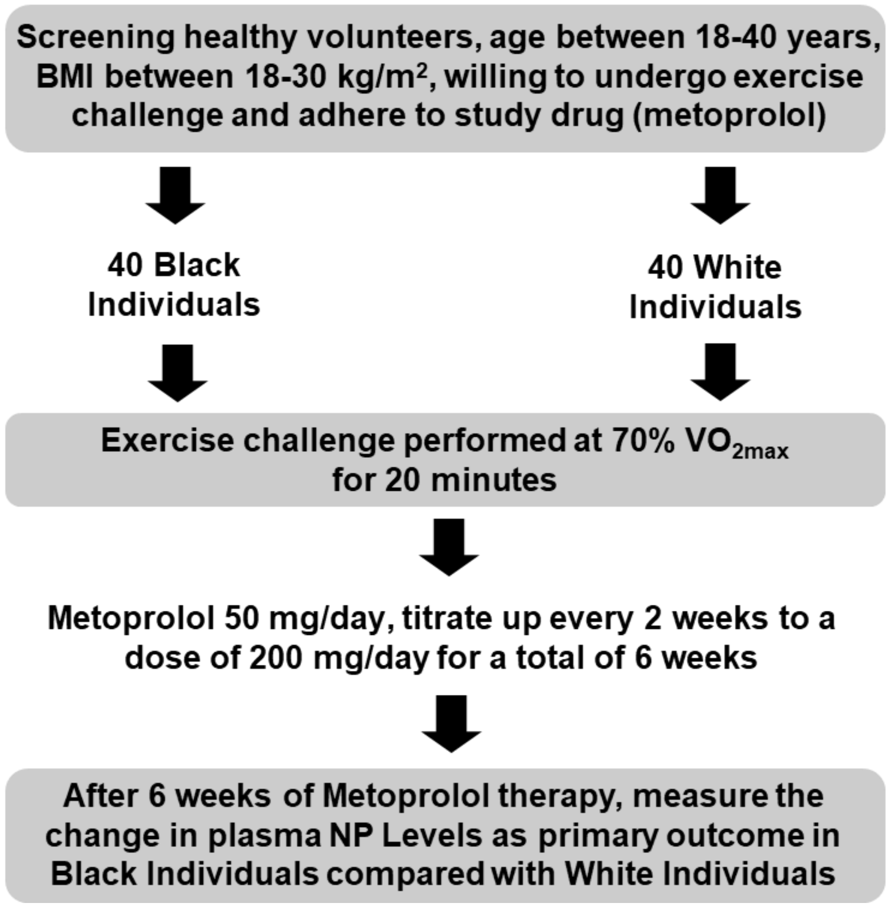

Procedures	Screening Visit	Exercise Capacity Determination Visit *Within 7 Days after Screening Visit*	Exercise Challenge Visit *3 Days After Exercise Capacity Visit*	Drug Initiation Visit *3-7 Days After Exercise Challenge Visit*	Interim Visit 1 *2 Weeks After Drug Initiation Visit*	Interim Visit 2 *2 Weeks After Interim Visit 1*	End of Study Visit *2 Weeks After Interim Visit 2*
**Written Informed Consent**	X						
**Participant Demography**	X						
**Medical History**	X	X	X	X	X	X	X
**Inclusion/Exclusion Criteria**	X	X					
**Concomitant Medications**	X	X	X	X	X	X	X
**Vital Signs (Blood Pressure/Heart Rate)**	X	X	X	X	X	X	X
**Exercise Questionnaire**	X						
**Adverse Events**					X	X	X
**Non-Fasting Blood Draw**	X						
**Fasting Blood Draw**			X	X	X	X	X
**24-Hour Urine Collection (Central Laboratory)**			X	X			X
**Spot Urine Collection**	X		X				
**Metoprolol Dispensation**				X	X	X	
**Assess Compliance**					X	X	X
**Telephonic Compliance/Side Effects Assessment (1 Week After Visit)**				X	X	X	
**Participant Compensation**	X	X	X	X	X	X	X

Study Outcomes

The primary endpoint in this study is the change in the NP levels, including NT-proBNP, BNP, and MR-proANP, in response to 6 weeks of metoprolol between young, healthy Black and White individuals. As the assessment of each NP is not independent of each other, we did not use the statistical correction of multiple testing.

The secondary outcome of this study was the change in the NP levels, including NT-proBNP, BNP, and MR-proANP, in response to a standardized exercise challenge between young, healthy

Black and White individuals.

Statistical Analysis

All analyses will be conducted using SAS 9.4 (Cary, NC) and the latest version of R Statistical Software (R core team 2020). Participants' demographic and baseline clinic characteristics will be summarized with mean and standard deviations (SD) for continuous variables and frequency and proportion for categorical variables. The 95% confidence interval (95%CI) will also be provided where appropriate. Statistical plots (scatters, histograms, Q-Q, bar charts, etc.) will be used to examine the distribution of data. Extreme values will be identified for validation or correction. The baseline differences between groups will be compared using a two-sample t-test for continuous variables or a Chi-square test for categorical variables. A nonparametric Wilcoxon test will be applied for skewed continuous data. For the primary and secondary outcomes, we will conduct a complete case analysis. The analysis will be conducted in individuals who completed both phases of the study i.e., exercise and medication. A sensitivity analysis will be conducted, including participants who completed a single phase (medication or exercise) of the study. All the tests will be two-sided with a significance level of 0.05.

Multivariable linear mixed models using change in log-transformed NP levels as the dependent variable and race as the independent variable (white will be chosen as reference) will be employed. Participants were taken as random effects. The fixed effects included race, time, a multiplicative interaction term of time and race, age, sex, body mass index, and insulin levels. Percentage difference and 95% confidence intervals in plasma NP levels after 6 weeks of metoprolol by race was calculated: ($[e^{\beta}–1]\times100$ multiplied by 6 weeks [where β is the beta coefficient from linear regression]).

Power Analysis

In the proposed study, we aim to recruit 40 Black individuals and 40 White individuals. **Supplementary Figure 11** presents the power estimates for the detectable mean fold-change in log NT-proBNP levels between groups (Black vs. White) from pre to post-6 weeks of metoprolol at a two-sided p-value of 0.05. In a prior study evaluating NP response after 6 weeks of metoprolol (similar dose and protocol), the mean ± SD relative change (measured in log units) in NT-proBNP levels was 0.66± 0.45. Based on preliminary data from 5 subjects who have already completed the proposed study, we have measured NPs and found a 110% increase in NT-proBNP levels after 6 weeks of metoprolol. As shown, we have excellent power to detect relatively small-medium differences (28%) between Black individuals and White individuals pre- and post- metoprolol. Even if we reduce the sample size by 20% (32 in each group) due to loss to follow- up or withdrawal, the detectable difference changes minimally, from 28% to 32%. **Supplementary Figure 12** depicts the relationship between power and effect size at a two-sided p-value of 0.05.

For the secondary outcome, we anticipate 80% power to detect a 39% (medium effect size) difference between groups (Black vs. White) in mean fold-change in BNP from before and after exercise challenge using the two-sided P value of 0.05. This is based upon data from Barletta et al. in which the mean ± SD relative change (measured in log units) in BNP between pre- and post- exercise in white individuals was 0.42 ± 0.61.[79]

Potential Risks

General. Risks related to overnight fasting include hypovolemia. Subjects will be instructed to drink plenty of fluids the day before the visit, and will have baseline blood pressure measured upon arrival at the clinical research unit (CRU). Risks related to venous blood draw include hematoma formation and phlebitis. This is a routine procedure that is considered standard of care in clinical medicine. At each visit, subjects will undergo a single venous blood draw with removal of approximately 4 teaspoons (20 ml) of blood. All blood draws will be performed by trained personnel using universal precautions to protect both the subject and personnel. The risks to subjects are minimal, but may include pain, allergic reaction, infection, or bleeding at the needle stick site. These usually resolve without any specific medical therapy over the course of minutes to days.

Tolerability and safety of study drug (Metoprolol). Common side effects reported in 3% or more cases of metoprolol use in the literature include dizziness, fatigue, and depression, shortness of breath, pruritus and diarrhea. Less frequent but serious side effects include wheezing, bronchospasm, bradyarrythmia and heart failure. However, since we are enrolling healthy volunteers the frequency of any side effects is less than 1%. All subjects will be monitored for adverse effects and during the study visits a physician will be present to monitor the safety of the subject through the protocol and between study visits, a physician will be available 24 hours a day by phone and/or pager to subjects. Metoprolol will be discontinued for any systolic blood pressure< 80 mm Hg and heart rate < 50 beats/minute at any visit. If the subject develops intolerance or any side effects from increasing the dose of metoprolol, they will go back to the maximally tolerated last dose to finish the 6-week duration.

Tolerability and safety of determination of VO_2 max and performing exercise challenge. The risks associated with the proposed study are minimal since moderate amounts of exercise generally promote good health, not endanger. Our eligibility criteria are established to exclude individuals for whom exercise is not appropriate. However, there is still moderate to high likelihood of fatigue, muscle soreness, or shortness of breath during and/or after performance of VO_2 max. There is an extremely low likelihood of injury to muscle, joint, ligaments, tendons or bone, tripping or falling, feeling faint or dizzy, chest pain, arrhythmia, and inconvenience. Furthermore, there is a very rare risk of cardiac ischemia or arrest during the peak VO_2 test or exercise challenge.

Tolerability and safety of standardized diets. While there is theoretic risk of eliciting hypertensive or hypotensive responses with standardized diets, Dr. Arora and the study team have decades of experience giving diets and have demonstrated that the standardized diets are well tolerated and safe.

Device complications. Not applicable

Psychosocial risks. We do not anticipate psychosocial risks to the subjects from participation in the proposed protocol. Strict confidentiality will be maintained to the fullest extent by the research team, including keeping all data in a secure, locked cabinet with limited access. All specimens and files will be coded after they are obtained and the code key kept in a locked cabinet.

Radiation risks. No additional radiation exposure will be required by the protocol.

Financial and legal risks. Those are felt to be minimal given the lack of expected clinical

information relevant to employment, insurability or paternity.

*Private health information***.** This information will be collected during the course of the study. However, only key study personnel will have access to this information, which will be stored in a HIPAA compliant, password protected REDCap database. No protected health information will be shared with employers, insurers, or non-research personnel.

Protection Against Risk

*Bodily fluids and biohazards***.** As phlebotomy for venous blood samples and urine collections will be performed, standard universal precautions will be utilized to protect both subjects and members of the research team. If discomfort, swelling, infection, or bleeding occurs in relation to the venous blood draw, appropriate medical therapy will be provided under the direction of the supervising physician. Most symptoms or side effects related to venous blood draw are temporary, self-resolving, and do not require any specific medical therapy.

*Data Protection and prompt reporting of adverse events***.** The University of Alabama at Birmingham regularly reviews its policies and procedures regarding the confidentiality of study subjects. All staff receive training on the critical importance of maintaining confidentiality. Data are stored and analyzed according to subject identification numbers, and in no instances are subjects' names or other identifying information available in publicly accessible files or in published material. Every effort is made to keep such data from being accessed inappropriately, but this possibility always remains, however remote. UAB Hospital has 24-hour security to protect all participants' data.

Location. The study protocol will be conducted in the UCEM and the UAB CRU (within the hospital with all available emergency medical personnel) under the direct supervision of a study physician. Portable defibrillators are present, and all staff members are certified for their use and CPR. Appropriate reporting, as described below, will be done in the event of an adverse event. Informed consent forms, case report forms, and data will be reviewed by the principal investigator and an independent data and safety monitor (details below) following enrollment of every 10 patients to ensure completeness, accuracy, and adherence to the protocol. Data to be reviewed includes appropriate handling and processing of blood samples and maintenance of patient confidentiality throughout the study. Specifically, for the interventional study, serious adverse events will be reported by phone, fax or e-mail within 24 hours to the UAB Human Research Committee, followed by a full written report within 10 working days/14 calendar days. Mild or moderate adverse events will be presented in progress reports at continuing reviews.

Potential for Benefit to Subjects and Others

*It is not anticipated that the participants of the study will directly benefit from the proposed research***.** However, the potential benefit to society is great. As hypertension and heart failure are common, particularly among Black individuals, the results of the proposed studies may provide important knowledge regarding the biological mechanisms and inform novel preventive and therapeutic strategies to be tested in future trials.

Monitoring and Quality Assurance

The risks associated with the study assessments are minimal. However, we will institute a three-member Data and Safety Monitoring Board (DSMB), comprised of experts in cardiology (Dr. David A. Calhoun, MD), biostatistics (Charity J. Morgan, Ph.D.), and pulmonary medicine (Dr. Surya P. Bhatt, MD), who will be charged with reviewing data and safety throughout the study, in addition to the Principal Investigator. Informed consent, case report forms and data will be reviewed by the principal investigator and DSMB. The DSMB will meet at least twice a year, either by phone or in person, to ensure the safety of study participants. The DSMB will be provided access to blinded safety data, including adverse events (AEs) by frequency and by body system, and study withdrawals following enrollment of every 10 patients to ensure completeness, accuracy, and adherence to the protocol. Other data to be reviewed include appropriate handling and processing of blood samples, laboratory data, and maintenance of patient confidentiality.

Safety monitoring will be performed throughout the course of the study. During and between all study visits and procedures, Dr. Arora will be available to address subject-related medical questions and ensure subject safety. At every subject visit, a study physician will carefully screen for any side effects from the study drug, record vital signs and monitor compliance. Any study-related injuries will be promptly addressed and treated using resources available at UAB.

All data will be recorded and stored in password-protected REDCap databases that can only be accessed by key study personnel. Data integrity and quality will be checked at the time of acquisition and entry. Prior to analyses, queries regarding missing data or extreme values will be addressed by checking against case report forms. Data handling and analytic methods will be recorded to ensure reproducibility of results. Results, when reported in any form, will be summarized without any patient identification. Standards for Personal Health Information will be followed to assure confidentiality.

Adverse Events (AE). The investigator or study physician will be responsible for detecting, documenting and reporting events that meet the definition of an AE. All Serious AEs (SAEs) and others will be collected from the time of the first dose of metoprolol through the final study visit. All-cause mortality and SAEs will be reported to the DSMB, and the local regulatory authority within 24 hours by the study monitor (principal investigator and/or study physician at UAB).

Definition of an AE. Any untoward or unfavorable medical occurrence in a participant, including any abnormal sign (for example, abnormal physical exam), symptom, or disease, temporally associated with the use of metoprolol, whether or not considered related to the participant's participation in the research. Three types of adverse event data will be reported: "All-Cause Mortality", "Serious", and "Other (Not Including Serious)" Adverse Events.

**All-Cause Mortality**	The occurrence of death due to any unrelated cause during the course of the study.
**Serious Adverse Events (SAEs)**	Bronchospasm (1%)
**Other (Not Including Serious) Adverse Events**	**Cardiovascular:** Bradycardia (3%), Hypotension (1%), Cold extremities (1%)<br>**Dermatologic:** Pruritus (5%), Rash (5%)<br>**Gastrointestinal:** Constipation (1%), Diarrhea (5%), and Indigestion (1%)<br>**Neurologic:** Dizziness (10%), Fatigue (10%), Headache<br>**Psychiatric:** Depression (5%)<br>**Respiratory:** Dyspnea (3%), Wheezing (1%)

Events meeting the definition of an AE include. New conditions detected or diagnosed after metoprolol administration, even though they may have been present prior to the start of the study. Signs, symptoms, or the clinical sequelae of a suspected interaction. Signs, symptoms, or the clinical sequelae of a suspected overdose of either metoprolol or concomitant medication (overdose per se will not be reported as SAEs and others).

Other Safety Assessment Abnormalities Reported as SAEs and others. Any abnormal safety assessments (e.g., vital sign measurements), including those that worsen from baseline and are felt to be clinically significant in the medical and scientific judgment of the investigator, are to be recorded as SAEs and others.

The review and decision regarding altering or stopping the protocol will be performed by the Principal Investigator, DSMB, and the study physician at UAB. Reasons to stop the study include an unexpected incidence of AEs attributed to metoprolol. The principal investigator will review any AEs immediately following their occurrence and report them to the Institutional Review Board (IRB) in accordance with their guidelines. All SAEs will be reported by phone, fax, or email within 24 hours to both the IRB and DSMB by the study monitor (principal investigator and/or study physician at UAB) followed by a full written report within 10 business/14 calendar days.

Supplementary Results

Sensitivity Analysis

The primary analysis was restricted to individuals completing the exercise and medication phase of the study. A sensitivity analysis was conducted utilizing all available data from participants completing either the exercise or the medication phase of the study. Data was available for 87 participants (44 White and 43 Black) in the exercise phase of the study and 83 participants (42 White and 41 Black) in the medication phase of the study.

Among 87 participants who completed the exercise phase of the study, MR-proANP levels increased by 39% (40 pg/mL at baseline to 56 pg/mL after exercise), BNP levels increased by 60% (15 pg/mL at baseline to 25 pg/mL after exercise), and NT-proBNP levels increased by 21% (23 pg/mL at baseline to 28 pg/mL after exercise) after exercise. Among 44 White participants, MR-proANP levels increased by 43% (42 pg/mL at baseline to 61 pg/mL immediately after exercise), BNP levels increased by 62% (15 pg/mL at baseline to 25 pg/mL immediately after exercise), and NT-proBNP was 22% (23 pg/mL at baseline to 28 pg/mL immediately after exercise). Among 43 Black participants, MR-proANP levels increased by 34% (38 pg/mL at baseline to 51 pg/mL immediately after exercise), BNP levels increased by 58% (15 pg/mL at baseline to 24 pg/mL immediately after exercise), and NT-proBNP levels increased by 10% (15 pg/mL at baseline to 17 pg/mL immediately after exercise). However, the increase in MR-proANP ($p_{interaction}$: 0.22) and BNP ($p_{interaction}$: 0.74) were similar across the race groups. The increase in NT-proBNP was higher in White individuals compared with Black individuals ($p_{interaction}$: 0.05). (**Supplementary Figure 9**)

Among 83 participants who completed the medication phase of the study, MR-proANP, BNP, and NT-proBNP levels increased by 17% (41 pg/mL at baseline to 48 pg/mL at 6 weeks),

59% (16 pg/mL at baseline to 25 pg/mL at 6 weeks), and 97% (19 pg/mL at baseline to 37 pg/mL at 6 weeks), respectively. Among 42 White participants, MR-proANP levels increased by 16% (44 pg/mL at baseline to 51 pg/mL at 6 weeks), BNP levels increased by 74% (15 pg/mL at baseline to 27 pg/mL at 6 weeks), and NT-proBNP levels increased by 99% (22 pg/mL at baseline to 45 pg/mL at 6 weeks). Among 41 Black participants, MR-proANP levels increased by 18% (39 pg/mL at baseline to 46 pg/mL at 6 weeks), BNP levels increased by 45% (16 pg/mL at baseline to 23 pg/mL at 6 weeks), and NT-proBNP levels increased by 95% (16 pg/mL at baseline to 31 pg/mL at 6 weeks). However, the increase in MR-proANP ($p_{interaction}$: 0.83), BNP ($p_{interaction}$: 0.24), and NT-proBNP ($p_{interaction}$: 0.97) was similar in Black and White participants. (**Supplementary Figure 10**)

Supplementary Figure 1. Study Population Flowchart

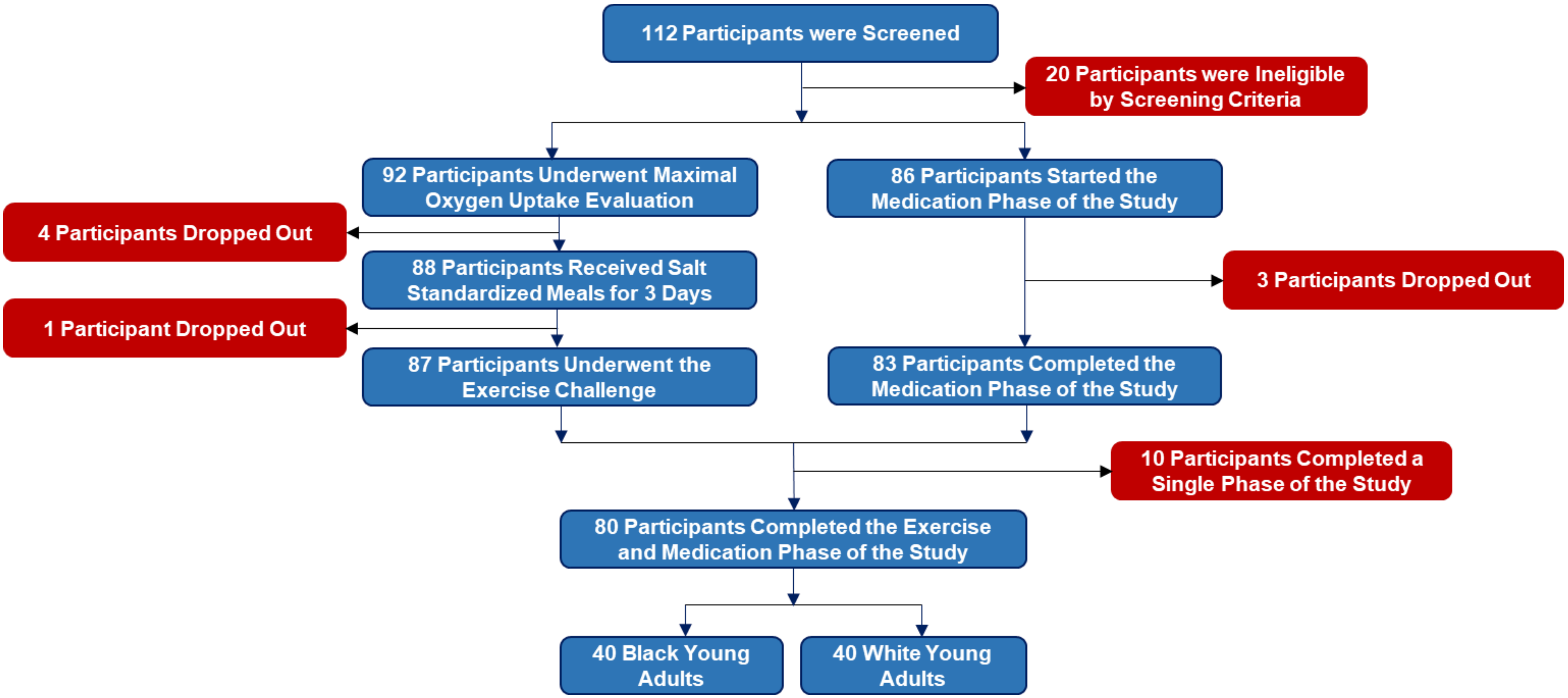

Supplementary Figure 2: Change in Least Square Means of Mid-Regional-pro Atrial Natriuretic Peptide, B-type Natriuretic Peptide, and N-Terminal-pro-B-Type Natriuretic Peptide in Response to a Standardized Exercise Challenge Stratified by Race

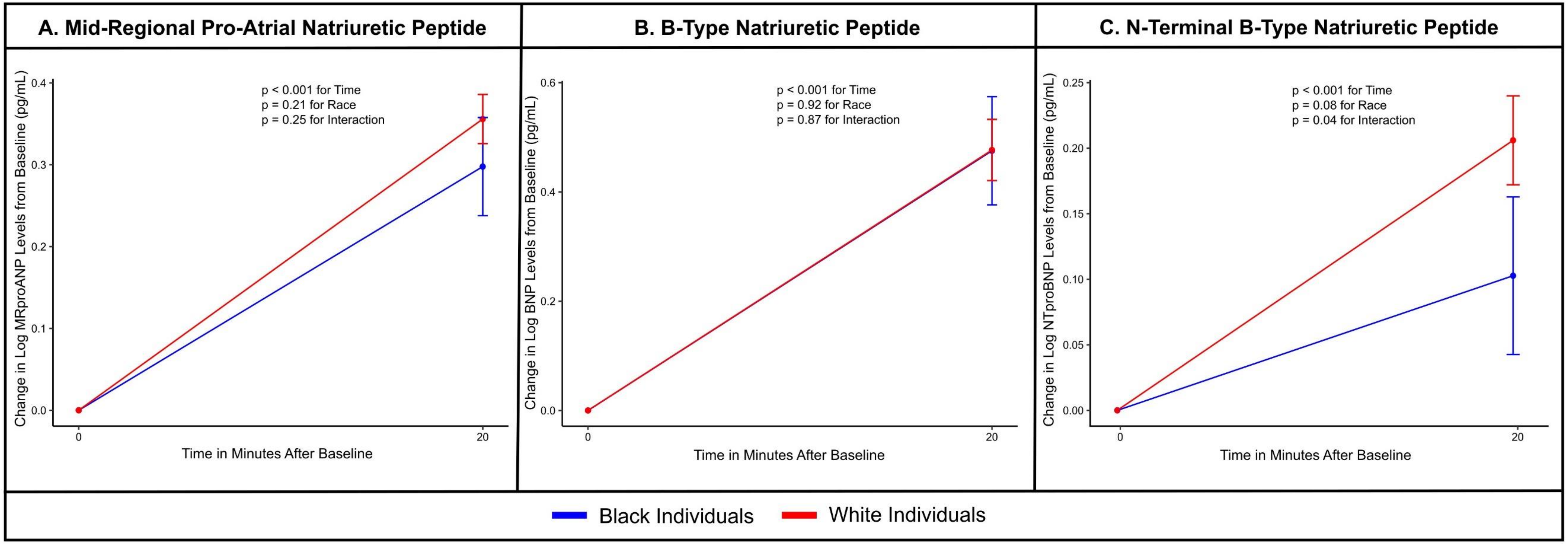

This figure depicts the change in mid-regional pro-atrial natriuretic peptide (A), B-type natriuretic peptide (B), and N-terminal proB-type natriuretic peptide (C) with standardized exercise stratified by race on the log scale. All participants underwent standardized exercise at 70% of their maximal oxygen uptake for 20 minutes. Natriuretic peptides were measured at baseline and immediately after exercise. Black (n=40) and White (n=40) individuals have been presented in blue and red, respectively. The values plotted depict the least square mean of natriuretic peptides and the standard error. Linear mixed models, adjusted for age, sex, body mass index, and serum insulin levels, were used to assess the racial differences in the natriuretic peptide response to standardized exercise. The two-sided p-values were derived from F-statistics and do not account for multiple comparisons.

Supplementary Figure 3: Change in Urinary Sodium Excretion with Standardized Exercise and 6 Weeks of Metoprolol Stratified by Race

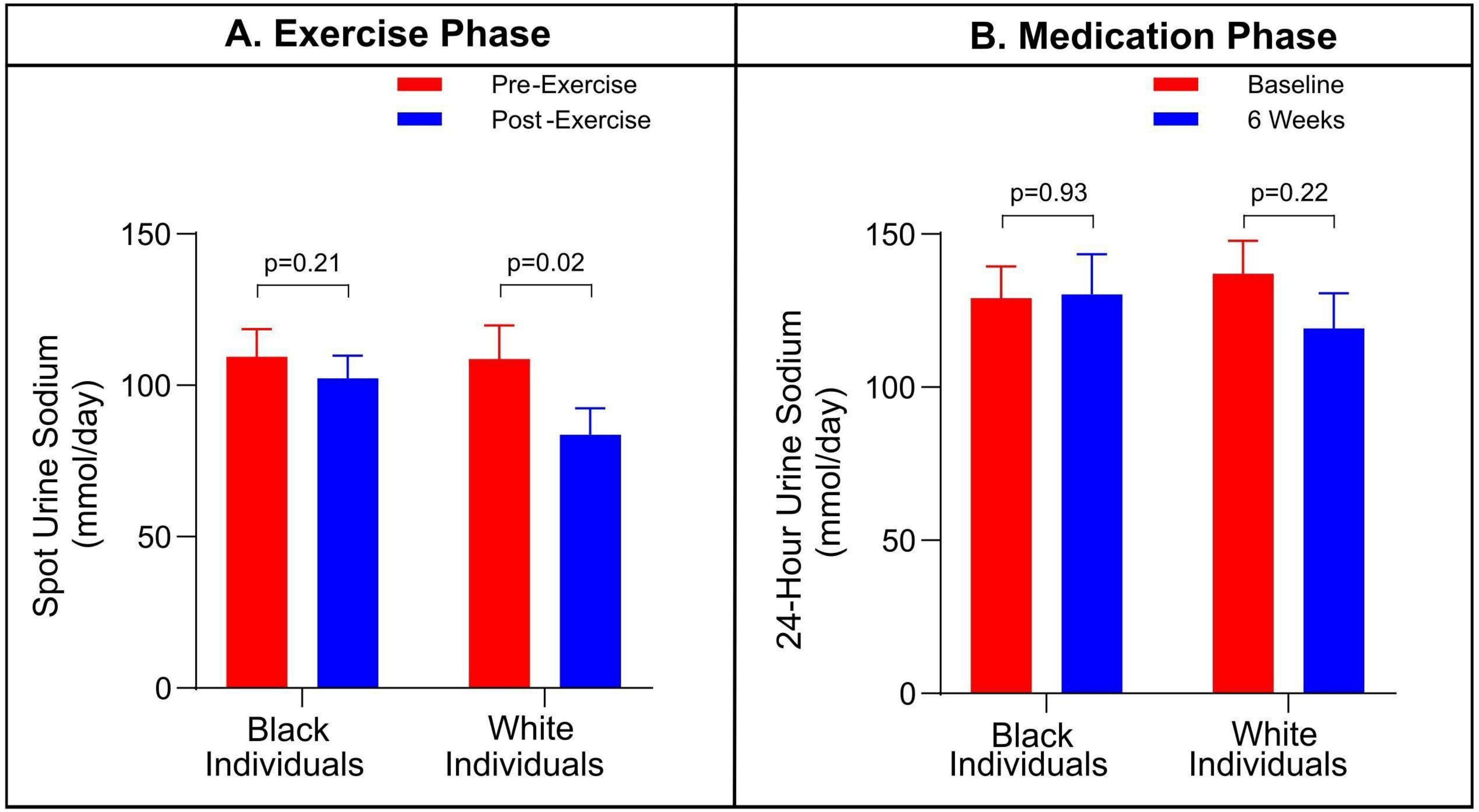

This figure depicts the change in urinary sodium levels collected during two phases of the study: the exercise and medication phases, stratified by race. During the exercise phase, spot urine samples were collected before and after standardized exercise for 20 minutes at 70% of maximal oxygen uptake, with data available for Black (n=28) and White (n=35) participants. During the medication phase, 24-hour urine samples were collected both before the initiation of metoprolol and after 6 weeks of therapy, with data available for Black (n=36) and White (n=34) participants. The values plotted depict the mean of urinary sodium levels and the standard error. Paired sample t-tests were used to assess within-group changes in urinary sodium levels before and after exercise and medication, stratified by race. The two-sided p-values were derived from the paired sample t-tests and do not account for multiple comparisons.

Supplementary Figure 4: Change in Blood Pressure and Heart Rate in Response to Exercise Stratified by Race

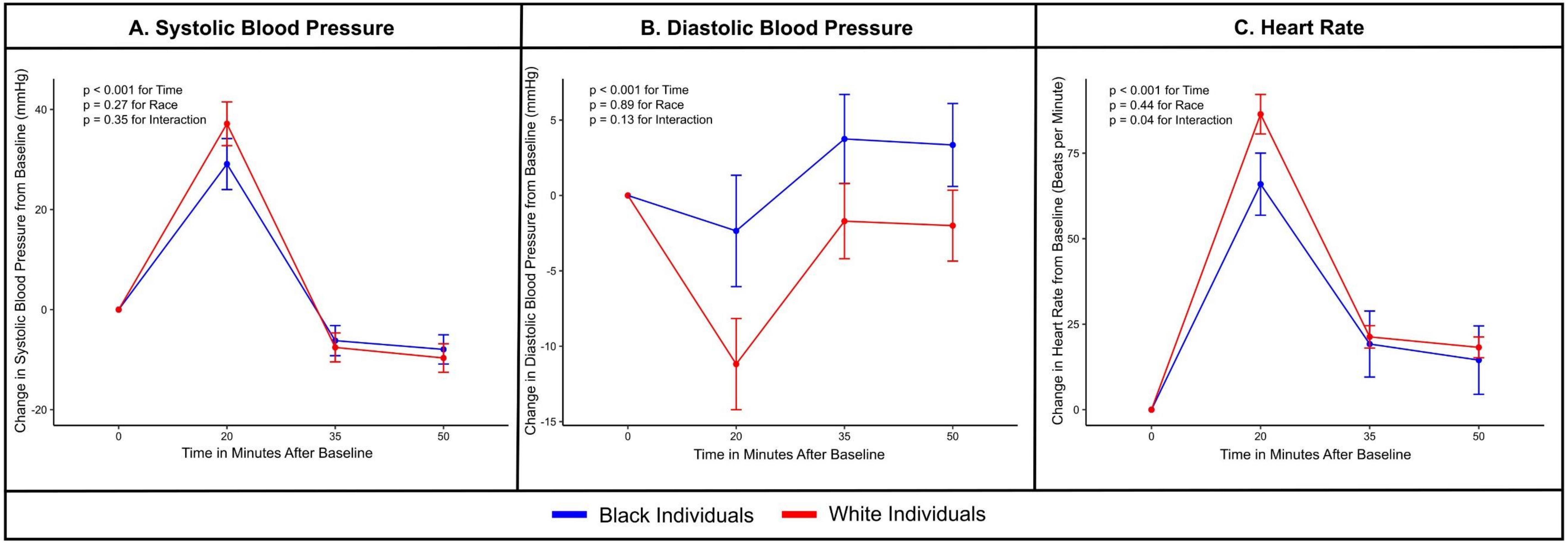

This figure depicts the systolic blood pressure (A), diastolic blood pressure (B), and heart rate (C) stratified by race during the exercise phase of the study. Measurements were obtained before, immediately, 15 minutes, and 30 minutes after exercise in each participant. The mean and standard error for each parameter has been depicted. Black and White individuals have been presented in blue and red, respectively. Data was available for 20 Black participants and 27 White participants. Linear mixed models, adjusted for age, sex, body mass index, and serum insulin levels, were used to assess the racial differences in the natriuretic peptide response to standardized exercise. The two-sided p-values were derived from F-statistics and do not account for multiple comparisons.

Supplementary Figure 5: Change in Least Square Means of Mid-Regional-pro Atrial Natriuretic Peptide, B-type Natriuretic Peptide, and N-Terminal-pro-B-Type Natriuretic Peptide in Response to 6 Weeks of Metoprolol Stratified by Race

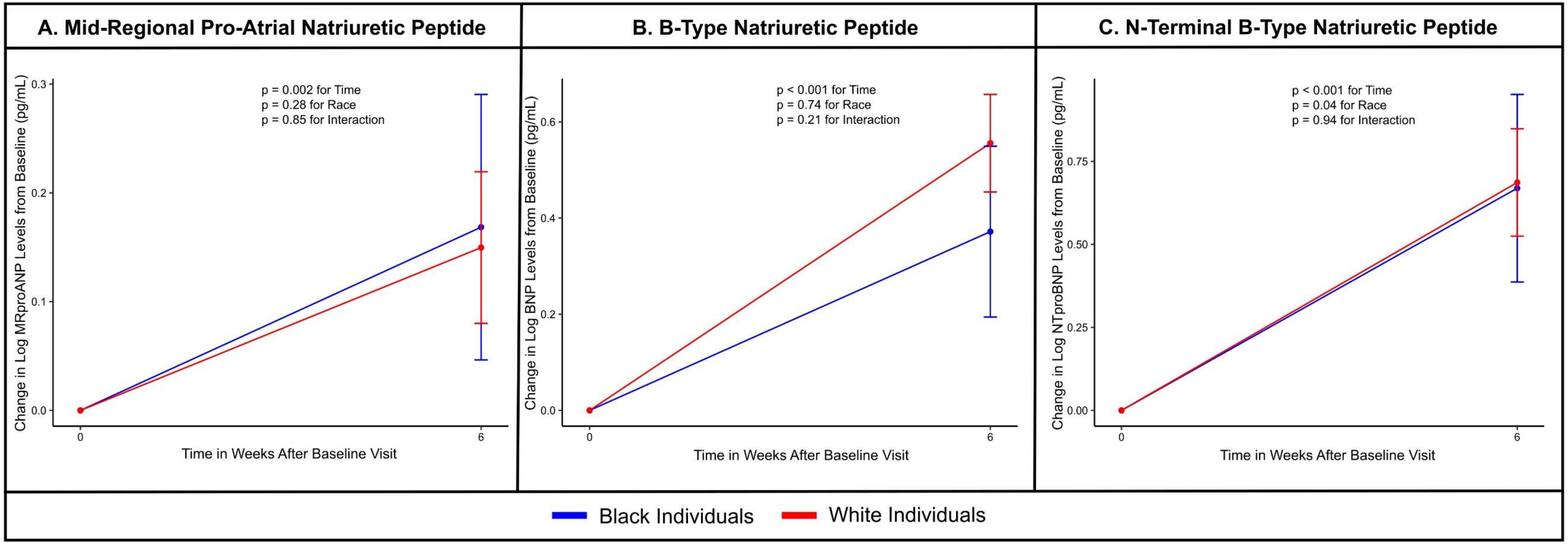

This figure depicts the change in mid-regional pro-atrial natriuretic peptide (A), B-type natriuretic peptide (B), N-terminal proB-type natriuretic peptide (C) with 6 weeks of metoprolol stratified by race on the log scale. Black (n=40) and White (n=40) individuals have been presented in blue and red, respectively. The values plotted depict the least square mean of natriuretic peptide and the standard error. Linear mixed models, adjusted for age, sex, body mass index, and serum insulin levels, were used to assess the racial differences in the natriuretic peptide response to metoprolol at 6 weeks. The two-sided p-values were derived from F-statistics and do not account for multiple comparisons.

Supplementary Figure 6: Change in Serum Insulin Levels in Response to a Standardized Exercise Challenge and 6 Weeks of Metoprolol Stratified by Race

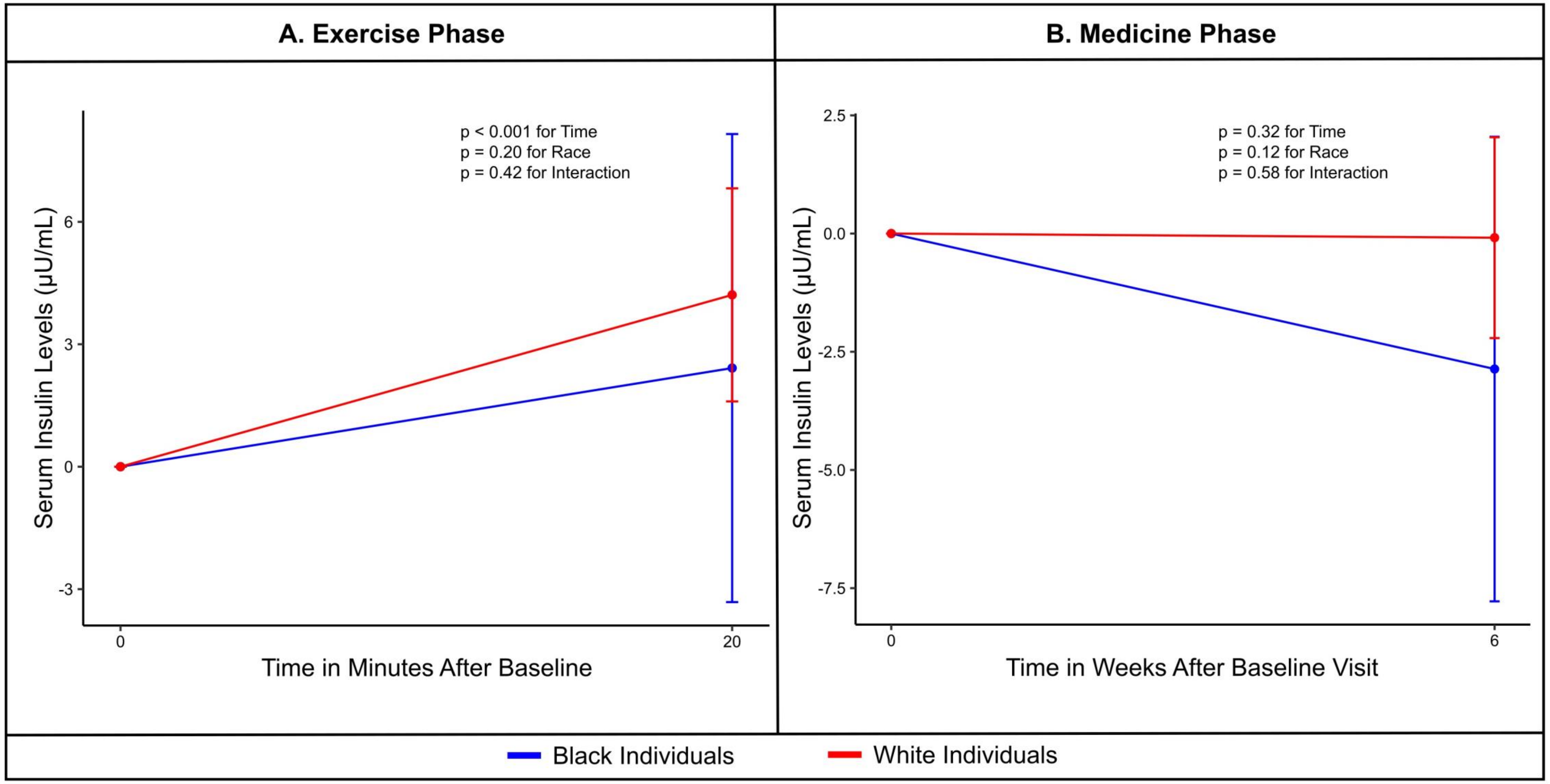

This figure depicts the change in serum insulin levels with standardized exercise and 6 weeks of metoprolol stratified by race. All participants underwent standardized exercise at 70% of their maximal oxygen uptake for 20 minutes. In the exercise phase, insulin levels were measured at baseline and immediately after exercise. In the medication phase, insulin levels were measured at baseline and at 6 weeks. Black (n=36) and White (n=40) individuals have been presented in blue and red, respectively. The values plotted depict the mean and the standard error. Linear mixed models, adjusted for age, sex, and body mass index were used to assess the racial differences in the serum insulin levels to metoprolol at 6 weeks. The two-sided p-values were derived from F-statistics and do not account for multiple comparisons.

Supplementary Figure 7: Change in Least Square Means of HOMA-IR in Response to 6 Weeks of Metoprolol Stratified by Race

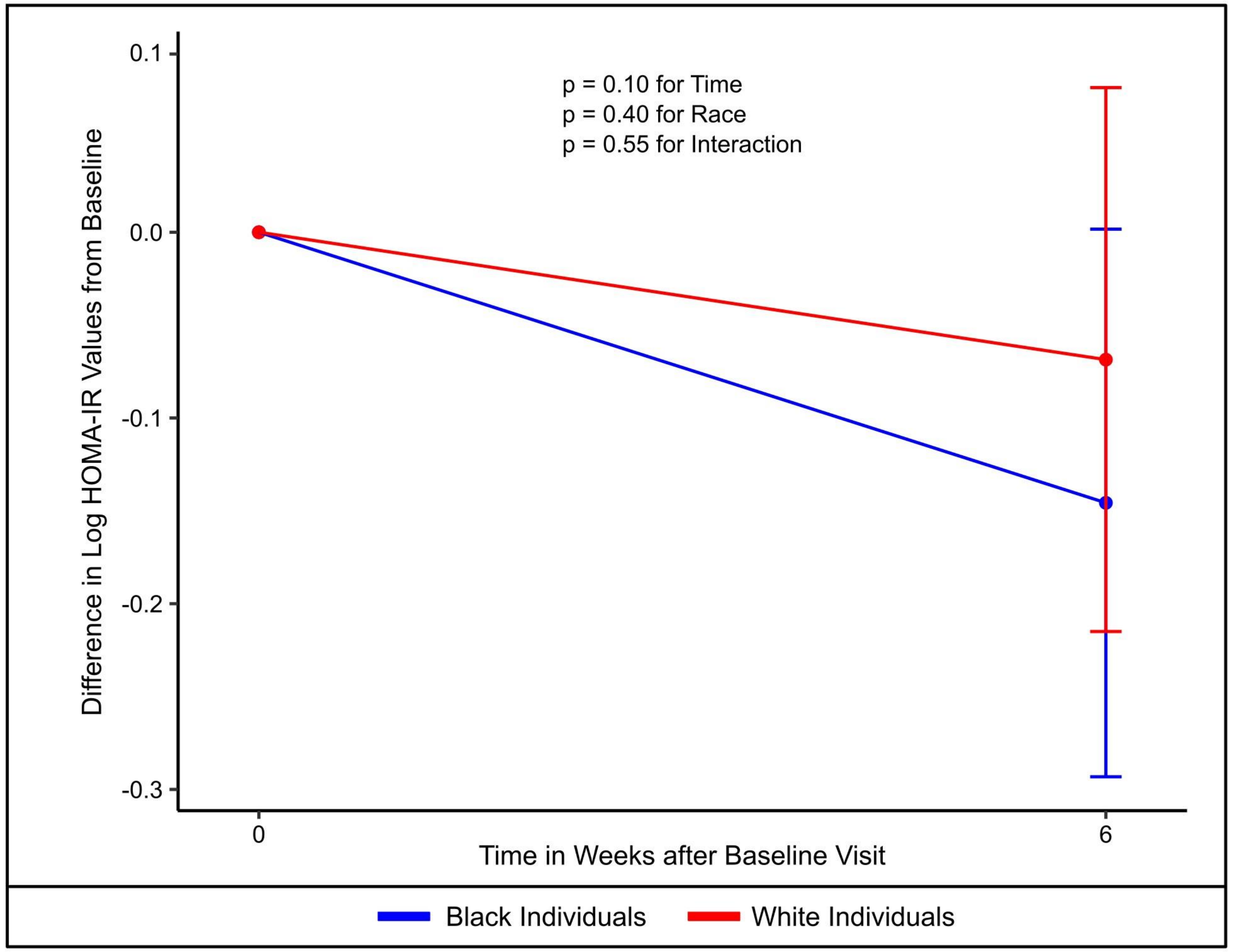

This figure depicts the change in HOMA-IR levels with 6 weeks of metoprolol stratified by race. HOMA-IR was calculated using glucose and insulin concentrations measured at baseline and at 6 weeks. Black (n=39) and White (n=40) individuals have been presented in blue and red, respectively. The values plotted represent the least square mean of HOMA-IR levels and the standard error on the log scale. Linear mixed models, adjusted for age, sex, and body mass index were used to assess racial differences in HOMA-IR in response to metoprolol at 6 weeks. The two-sided p-values were derived from F-statistics and do not account for multiple comparisons.

Supplementary Figure 8: Change in Least Square Means of Plasma Aldosterone Concentrations in Response to 6 Weeks of Metoprolol Stratified by Race

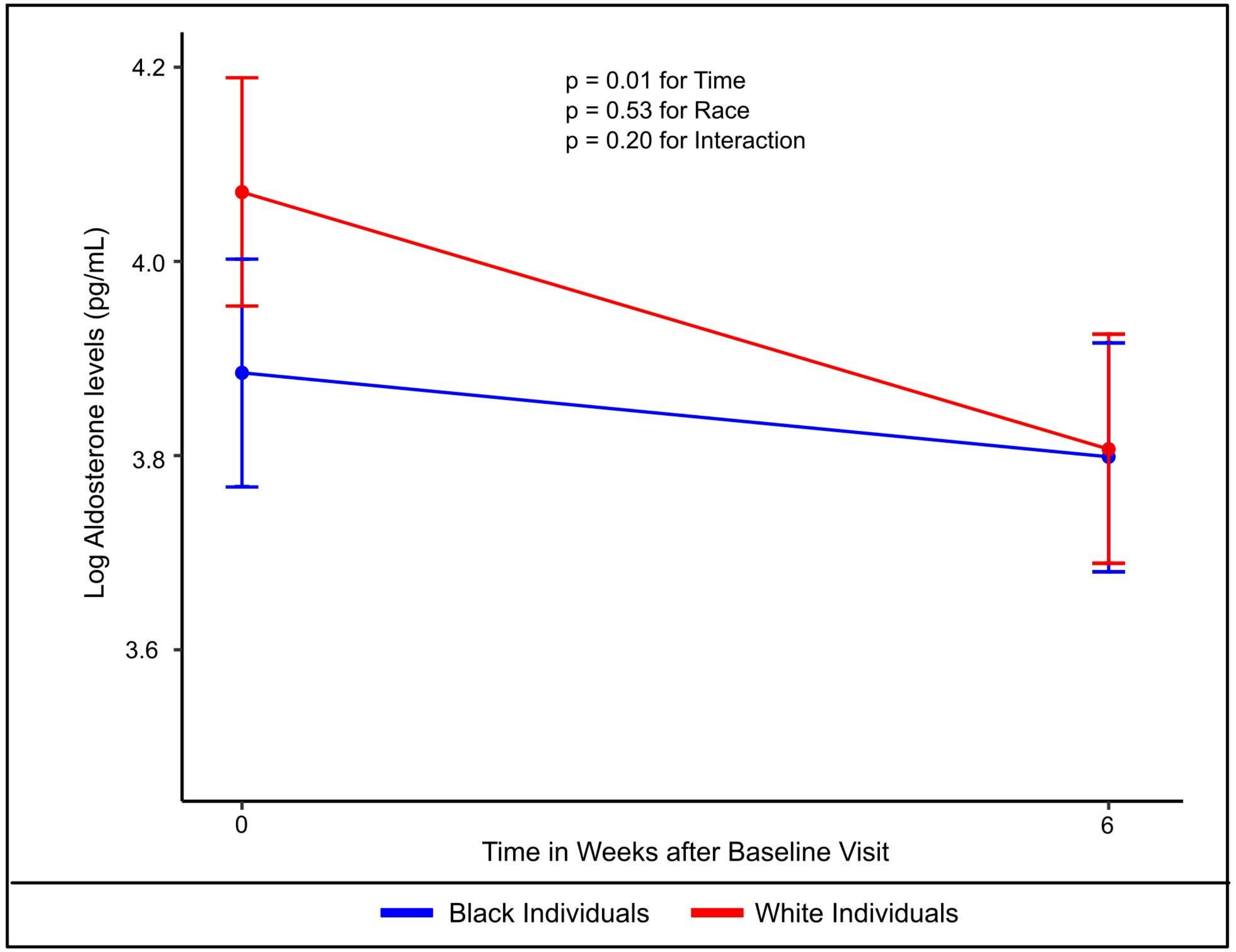

This figure depicts the change in plasma aldosterone concentrations after 6 weeks of metoprolol, stratified by race on the log scale. Black (n=39) and White(n=40) individuals are represented in blue and red, respectively. The values plotted represent the least square mean of plasma aldosterone concentrations and the standard error on the log scale. Linear mixed models, adjusted for age, sex, body mass index, and serum insulin levels, were used to assess racial differences in plasma aldosterone concentrations in response to metoprolol at 6 weeks. The two-sided p-values were derived from F-statistics and do not account for multiple comparisons.

Supplementary Figure 9: Sensitivity Analysis Assessing the Change in Mid-Regional-pro Atrial Natriuretic Peptide, B-type Natriuretic Peptide, and N-terminal-pro-B-type Natriuretic Peptide in Response to a Standardized Exercise Challenge Stratified by Race Including All Participants with Data from the Exercise Visit

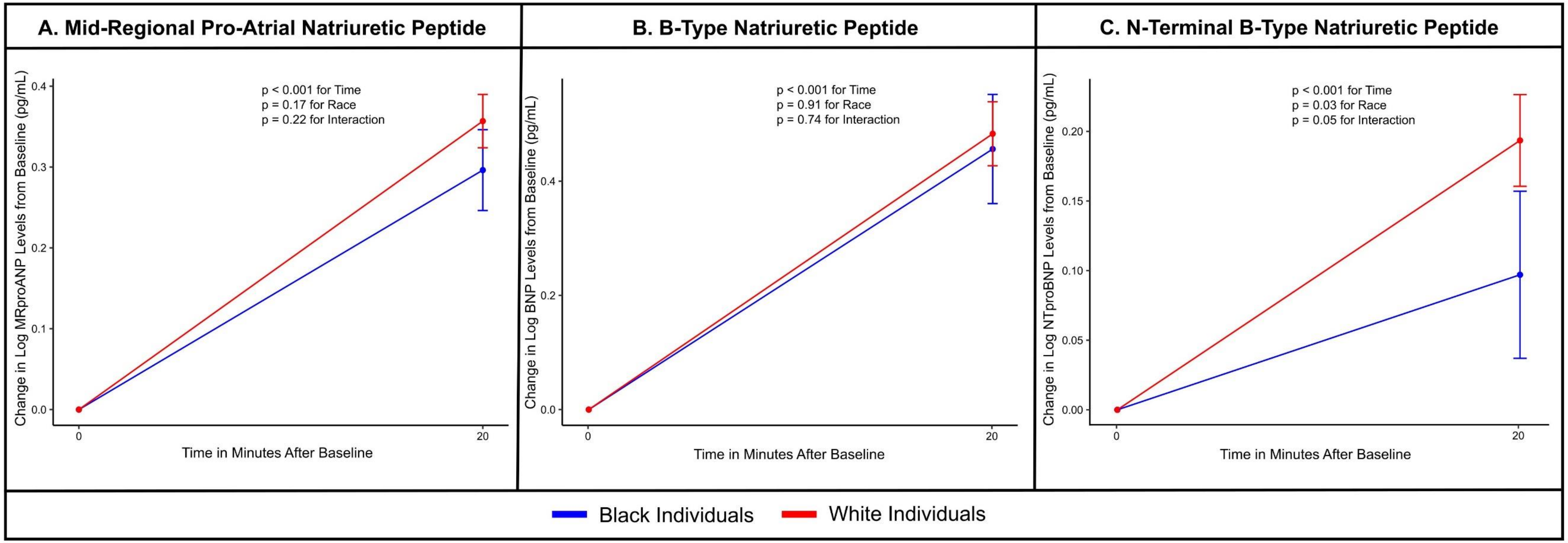

This figure depicts the change in mid-regional pro-atrial natriuretic peptide (A), B-type natriuretic peptide (B), and N-terminal proB-type natriuretic peptide (C) with standardized exercise stratified by race on the log scale. All participants underwent standardized exercise at 70% of their maximal oxygen uptake for 20 minutes. Natriuretic peptides were measured at baseline and immediately after exercise. Black (n=43) and White(n=44) individuals have been presented in blue and red, respectively. The values plotted depict the least square mean and the standard error. Linear mixed models, adjusted for age, sex, body mass index, and serum insulin levels, were used to assess the racial differences in the natriuretic peptide response to standardized exercise. The two-sided p-values were derived from F-statistics and do not account for multiple comparisons.

Supplementary Figure 10: Sensitivity Analysis Assessing the Change in Mid-Regional-pro Atrial Natriuretic Peptide, B-type Natriuretic Peptide, and N-terminal-pro-B-type Natriuretic Peptide in Response to a 6 Weeks of Metoprolol Stratified by Race Including All Participants with Data from the Medication Phase of the Study

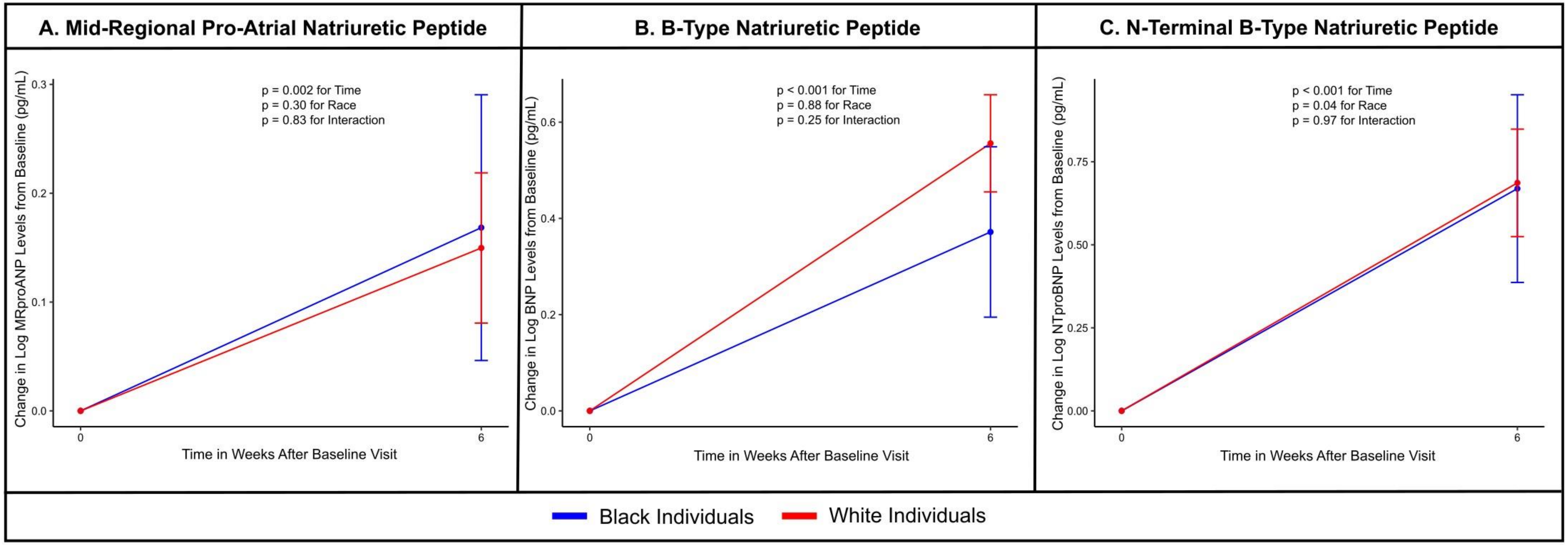

This figure depicts the change in mid-regional pro-atrial natriuretic peptide (A), B-type natriuretic peptide (B), N-terminal proB-type natriuretic peptide (C) with 6 weeks of metoprolol stratified by race on the log scale. Black and White individuals have been presented in blue and red, respectively. The values plotted depict the least square mean and the standard error. Linear mixed models, adjusted for age, sex, body mass index, and serum insulin levels, were used to assess the racial differences in the natriuretic peptide response to metoprolol at 6 weeks. The two-sided p-values were derived from F-statistics and do not account for multiple comparisons.

Supplementary Figure 11: Power Curve Showing the Relationship Between Power and Percentage Difference in N-terminal-pro-B-type Natriuretic Peptide Levels Between Black and White individuals from Pre-Treatment to 6 weeks Post-Metoprolol

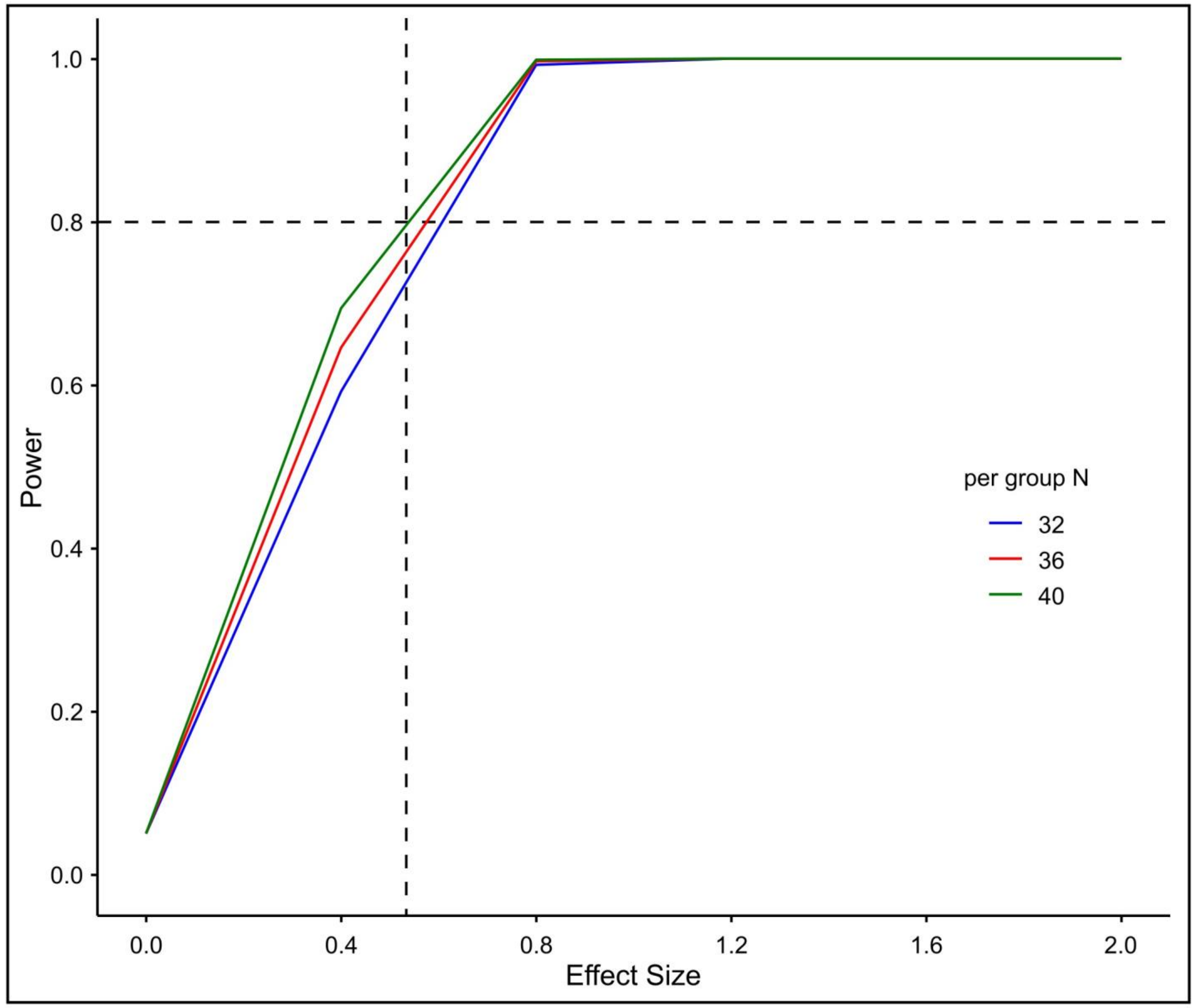

This figure depicts the power curve for detecting the percentage difference in the mean change in N-terminal pro-B type natriuretic peptide (NTproBNP) levels from pre-treatment to 6 weeks post-metoprolol between White and Black individuals. Power analysis was conducted for Black and White individuals, with per-group sample sizes of 32, 36, and 40 represented by blue, red, and green lines, respectively. The plotted values indicate power estimates across varying effect sizes, with dashed lines marking the 0.8 power threshold (horizontal) and a mean percentage difference of 28% (vertical).

Supplementary Figure 12: Power Curve Showing the Relationship Between Power and Effect Size between Black and White individuals from pre-treatment to 6 weeks post-Metoprolol

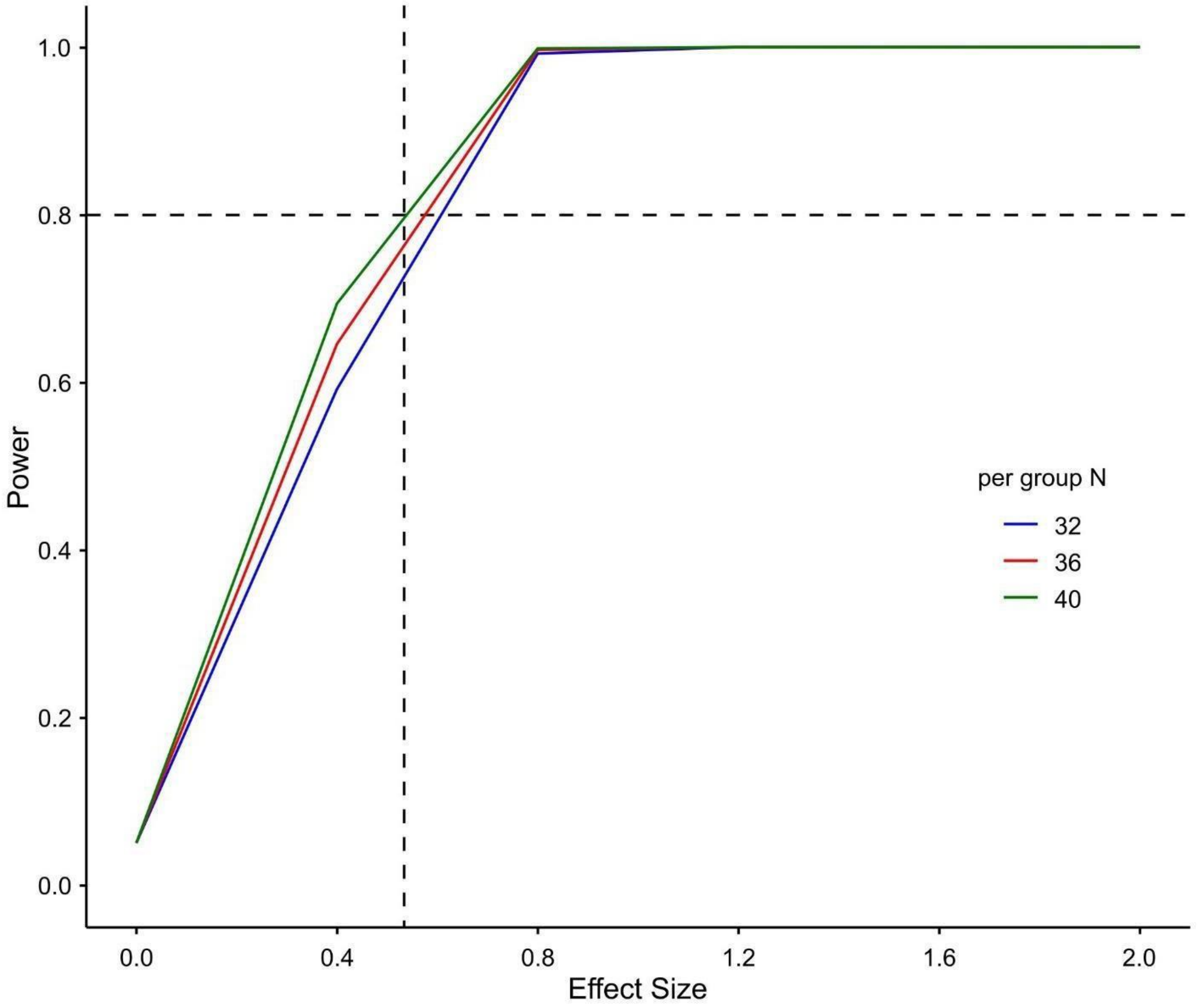

This figure depicts the power curve for detecting effect size of the difference in the change in N-terminal pro-B type natriuretic peptide (NT-proBNP) levels from pre-treatment to 6 weeks post-metoprolol between White and Black individuals. Power analysis was conducted for Black and White individuals, with per- group sample sizes of 32, 36, and 40 represented by blue, red, and green lines, respectively. The plotted values indicate power estimates across varying effect sizes, with dashed lines marking the 0.8 power threshold (horizontal) and an effect size of 0.53 (vertical).
