# Supplementary material for: Differences in natriuretic peptide response in self-identified white and black individuals: a physiological clinical trial
Source: Nat Commun. 2025 Feb 13;16:1621. doi: 10.1038/s41467-024-55648-2 (PMC11825667; doi:10.1038/s41467-024-55648-2)
Supplement: Supplementary file 2 — Reporting Summary [file 41467_2024_55648_MOESM2_ESM.pdf]

nature portfolio

Corresponding author(s): Pankaj Arora

Last updated by author(s): Aug 28, 2024

Reporting Summary

Nature Portfolio wishes to improve the reproducibility of the work that we publish. This form provides structure for consistency and transparency in reporting. For further information on Nature Portfolio policies, see our Editorial Policies and the Editorial Policy Checklist.

Statistics

For all statistical analyses, confirm that the following items are present in the figure legend, table legend, main text, or Methods section.

n/a	Confirmed	
☐	☒	The exact sample size (*n*) for each experimental group/condition, given as a discrete number and unit of measurement
☐	☒	A statement on whether measurements were taken from distinct samples or whether the same sample was measured repeatedly
☐	☒	The statistical test(s) used AND whether they are one- or two-sided<br>*Only common tests should be described solely by name; describe more complex techniques in the Methods section.*
☐	☒	A description of all covariates tested
☐	☒	A description of any assumptions or corrections, such as tests of normality and adjustment for multiple comparisons
☐	☒	A full description of the statistical parameters including central tendency (e.g. means) or other basic estimates (e.g. regression coefficient) AND variation (e.g. standard deviation) or associated estimates of uncertainty (e.g. confidence intervals)
☐	☒	For null hypothesis testing, the test statistic (e.g. *F*, *t*, *r*) with confidence intervals, effect sizes, degrees of freedom and *P* value noted<br>*Give P values as exact values whenever suitable.*
☒	☐	For Bayesian analysis, information on the choice of priors and Markov chain Monte Carlo settings
☒	☐	For hierarchical and complex designs, identification of the appropriate level for tests and full reporting of outcomes
☒	☐	Estimates of effect sizes (e.g. Cohen's *d*, Pearson's *r*), indicating how they were calculated

Our web collection on statistics for biologists contains articles on many of the points above.

Software and code

Policy information about availability of computer code

Data collection	It is a a single-center, prospective clinical trial (NCT# NCT03070184) conducted from 2018 to 2023 at the University of Alabama at Birmingham (UAB) in United States of America. The detailed description of data collection and study procedure was outlined in the study protocol.
Data analysis	All analysis was conducted on SAS 9.4 (Cary, NC). The detailed description was provided in the statistical analysis.

For manuscripts utilizing custom algorithms or software that are central to the research but not yet described in published literature, software must be made available to editors and reviewers. We strongly encourage code deposition in a community repository (e.g. GitHub). See the Nature Portfolio guidelines for submitting code & software for further information.

Data

Policy information about availability of data

All manuscripts must include a data availability statement. This statement should provide the following information, where applicable:

- Accession codes, unique identifiers, or web links for publicly available datasets
- A description of any restrictions on data availability
- For clinical datasets or third party data, please ensure that the statement adheres to our policy

Data from this study are available upon reasonable request. Interested parties should contact the corresponding author for access to the data.

Research involving human participants, their data, or biological material

Policy information about studies with human participants or human data. See also policy information about sex, gender (identity/presentation), and sexual orientation and race, ethnicity and racism.

Reporting on sex and gender	This study used self-reported sex to define baseline characteristics and as a covariate in the statistical models.
Reporting on race, ethnicity, or other socially relevant groupings	Self-reported race and ethnicity were used to define baseline characteristics and performed race-stratified models.
Population characteristics	This study included 80 individuals (40 Whites, 40 Blacks). The median age was ~26 years with ~50% females.
Recruitment	This study included self-identified Black and White individuals between 18 to 40 years of age with a body mass index between 18 to 30 kg/m2, seated blood pressure <140/90 mm Hg, who were willing to take the study medication and were able to exercise. Detailed description of participant recruitment outlined in the study protocol.
Ethics oversight	The trial was approved by the UAB Institutional Review Board (IRB#: 170214001)

Note that full information on the approval of the study protocol must also be provided in the manuscript.

Field-specific reporting

Please select the one below that is the best fit for your research. If you are not sure, read the appropriate sections before making your selection.

- [x] Life sciences
- [ ] Behavioural & social sciences
- [ ] Ecological, evolutionary & environmental sciences

For a reference copy of the document with all sections, see nature.com/documents/nr-reporting-summary-flat.pdf

Life sciences study design

All studies must disclose on these points even when the disclosure is negative.

Sample size	Total of 80 individuals (40 Whites, 40 Blacks). The sample size calculations for power was provided in the study.
Data exclusions	Participants were excluded based on predefined exclusion criteria. The exclusion criteria included decreased renal function (estimated glomerular filtration rate <60 mL/min/1.73m2), history of cardiovascular disease, history of diabetes, history of hypertension, anemia, pregnancy, use of hormone replacement therapy/oral contraceptives, depression, low systolic blood pressure (<100 mm Hg) or diastolic blood pressure (<60 mm Hg) at screening, low heart rate at screening (<60/min) at screening, elevated liver function tests (>3x upper normal limit), and history of smoking. A detailed description of exclusion criteria is outlined in the study protocol.
Replication	All the study outcomes i.e., plasma natriuretic pepetide concentrations were measured on automated platforms with high reproducibility. A detailed description of laboratory measurements are outlined in the study protocol.
Randomization	This study was prospective study by self-identified race. Randomization was not performed.
Blinding	This study include a specific intervention by race. Blinding was not performed during the recruitment as well as statistical analyses. However, laboratory personnels were blinded to study participants while performing assessments.

Reporting for specific materials, systems and methods

We require information from authors about some types of materials, experimental systems and methods used in many studies. Here, indicate whether each material, system or method listed is relevant to your study. If you are not sure if a list item applies to your research, read the appropriate section before selecting a response.

Materials & experimental systems

n/a	Involved in the study	
[x]	[ ]	Antibodies
[x]	[ ]	Eukaryotic cell lines
[x]	[ ]	Palaeontology and archaeology
[x]	[ ]	Animals and other organisms
[ ]	[x]	Clinical data
[x]	[ ]	Dual use research of concern
[x]	[ ]	Plants

Methods

n/a	Involved in the study	
[x]	[ ]	ChIP-seq
[x]	[ ]	Flow cytometry
[x]	[ ]	MRI-based neuroimaging

Clinical data

Policy information about clinical studies

All manuscripts should comply with the ICMJE guidelines for publication of clinical research and a completed CONSORT checklist must be included with all submissions.

Clinical trial registration	NCT#03070184
Study protocol	The full protocol has been provided as a supplementary file.
Data collection	Healthy participants were recruited from the UAB Campus and the Birmingham metropolitan area between 2018 and 2023.
Outcomes	The primary outcome of the study was the change in the plasma NP concentrations, including NT-proBNP, BNP, and MR-proANP, in response to 6 weeks of metoprolol between young, healthy black and white individuals. The secondary outcomes included the change in plasma NP concentrations, including NT-proBNP, BNP, and MR-proANP, immediately after exercise between Black and White individuals.

Plants

Seed stocks	NA
Novel plant genotypes	NA
Authentication	NA
